# A novel integrated platform for the identification of surgical margins in oral squamous cell carcinoma: results from a prospective single-institution series

**DOI:** 10.1186/s12885-019-5634-0

**Published:** 2019-05-17

**Authors:** Alessandro Baj, Nicola Fusco, Alessandro Bolzoni, Daniela Carioli, Camilla Mazzucato, Alice Faversani, Lorenzo Bresciani, Marco Maggioni, Pasquale Capaccio

**Affiliations:** 1 Department of Biomedical, Surgical, and Dental Sciences, University of Milan, Fondazione IRCCS Ca’ Granda Policlinico, via Francesco Sforza, 35, 20122 Milan, Italy; 2 Maxillo-Facial Surgery and Odontostomatology Unit, Fondazione IRCCS Ca’ Granda Ospedale Maggiore Policlinico, Milan, Italy; 3 Division of Pathology, Fondazione IRCCS Ca’ Granda Ospedale Maggiore Policlinico, Milan, Italy; 4 Otolaryngology Unit, Fondazione IRCCS Ca’ Granda Ospedale Maggiore Policlinico, Milan, Italy

**Keywords:** Oral squamous cell carcinoma, NBI, Narrow band imaging, Microvascular density, Surgical margins

## Abstract

**Background:**

The optimal surgical margins assessment is capital in oral squamous cell carcinoma (OSCC) management. We evaluated the clinical benefits of integrating intraoperative macroscopic margin (MM) assessment and narrow band imaging (NBI).

**Methods:**

Sixteen OSCC patients eligible for surgery were prospectively enrolled. For each patient, 2 to 6 bioptic samples of MM and NBI margins were obtained and histologically analyzed for the presence of dysplasia and lymphocytes. Microvessel density was investigated by CD34 immunohistochemistry.

**Results:**

Taken together, 104 specimens were analyzed, including 15% tumors, 33% MM, 33% NBI margins, and 19% MM-NBI overlapping margins. The NBI margins were closer to the lesion in 50% cases, while the same number of MM were more conservative than NBI, irrespective of the tumor site. The rate of histologically positive margins was similar among the two methods, akin to the microvessel density.

**Conclusions:**

MM assessment should be integrated but not replaced with the NBI technology to allow for more conservative surgery.

## Background

Oral squamous cell carcinoma (OSCC) is the most frequent histological type of head and neck cancer and one of the most prevalent malignant neoplasms worldwide [1]. Despite the recent achievements in the diagnosis and treatment of these patients, OSCC is showing increasingly high recurrence rates [2, 3]. Due to its clinical and biological complexity, therapeutic decision-making is not an easy task, even in multidisciplinary settings. Anatomical site, clinical stage, and pathological features of the primary tumor are the foremost elements to guide OSCC treatment, which remains surgically-based either in single or in combined therapeutic settings [4]. During surgical removal, the visible neoplastic area should be resected with a threshold of normal tissue, whose edge represents the mucosal margin [5]. To improve patient’s outcome, the surgical radicality (i.e. histologically-proved negativity of the mucosal margin) is fundamental [6]. Indeed, there are multiple lines of evidence to suggest that failure to reach clear margins in OSCC is related to an increased risk of local recurrence and, subsequently, reduced chances of survival. However, there are no widely adopted guidelines for pre- and intra-operative margins identification. To date, finding the “golden strategy” for the optimal assessment of the surgical margins remains one of the most critical issues in OSCC management [7, 8].

Several approaches have been proposed to enhance the traditional white-light macroscopic margins (MM) identification in OSCC. Among them, the Narrow Band Imaging (NBI) technology have shown good performance and is currently employed in several Centers [9, 10]. This augmented reality tool increases the contrast between the epithelial surface and the subjacent vascular network, allowing for the visualization of the mucosal and submucosal (micro) vascular patterns. The principle by which NBI can be employed for surgical margin assessment is based on the evidence that neoangiogenesis is a crucial step in tumor growth and metastatic spread. Therefore, the in vivo analysis of blood-specific light traces could help identifying oral potentially malignant disorders or even overt malignant conditions at the periphery of the resected tumor. Furthermore, several studies have demonstrated that microvessel density (MD) assessed by histological and immunohistochemical analysis can be employed as a prognostic biomarker for OSCC [11–13].

Our work aims to evaluate the potential surgical benefits of mucosal margin assessment for OSCC using a platform which integrates intraoperative MM and NBI.

## Methods

### Patients and tissue specimens

This pilot prospective non-randomized study was approved by the local ethics committee (approval #19_2018bis). A total of 16 patients (10 males, 6 females) with OSCC eligible for surgical treatment were enrolled. Informed consent was obtained from all patients. Only patients diagnosed and managed in IRCCS Ca′ Granda Foundation – Policlinico Maggiore Hospital, Milan, Italy, > 18 years old, chemotherapy- and radiotherapy-naïve, with no history of cancer were included. All patients underwent surgical treatment with a program established according to the guidelines of the American Joint Committee on Cancer (7th edition) [2].

### Macroscopic and narrow band imaging surgical margins assessment in vivo

**Fig. 1:**
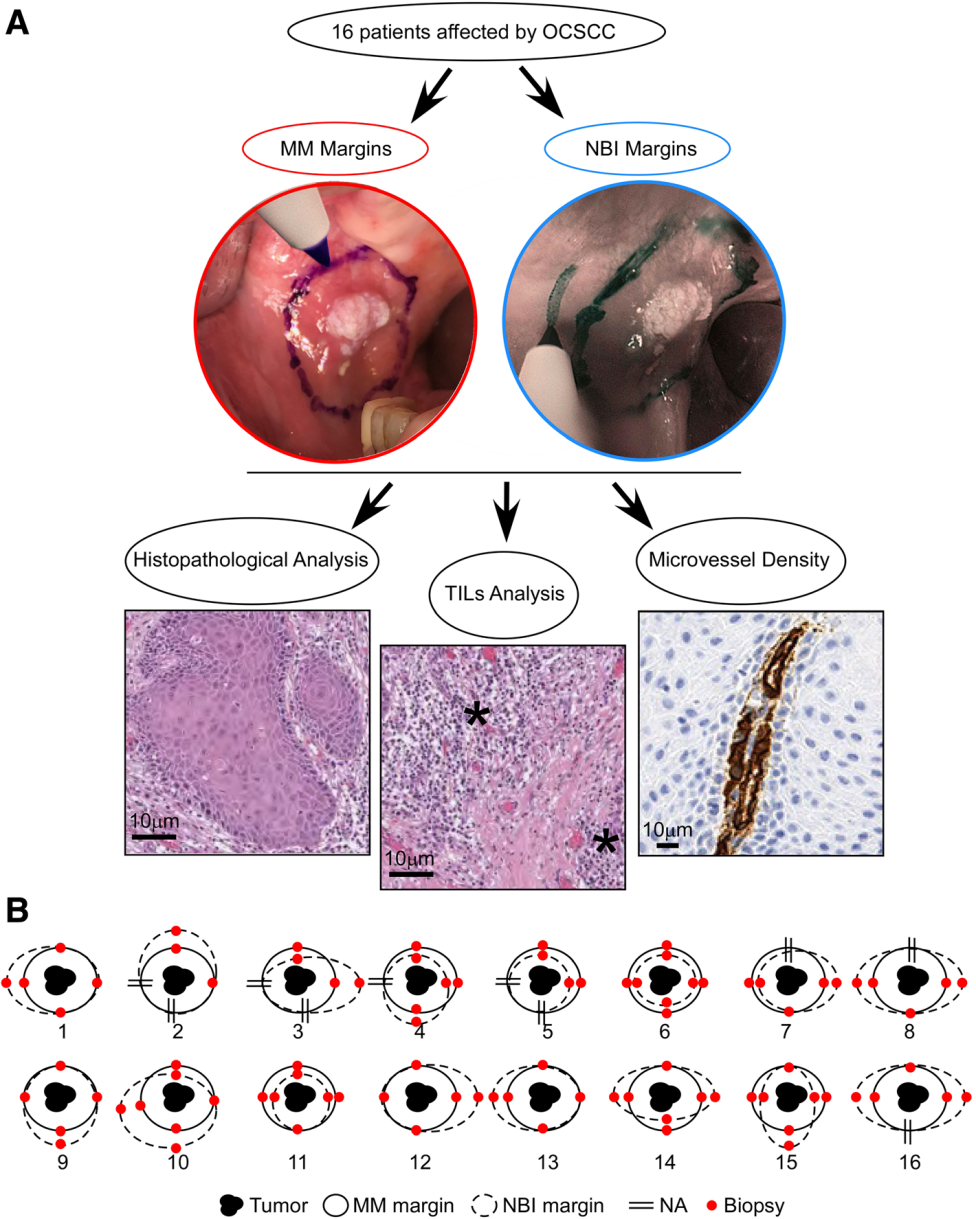
**a** Schematic overview of the study. TILs are highlighted by stars. Original magnification of the micrographs 100X. **b** Schematic image of MM and NBI margins for the 16 OSCC patients. Red dots represent the cardinal points related to the biopsies performed for the excision of the mucosal margins

The MM was assessed by a craniofacial surgeon at a distance of 1,5–2 cm from the tumor, as described (Fig. 1a) [7, 14]. Subsequently, two ears, nose and throat (ENT) surgeons performed intraoperative NBI endoscopic evaluation using a scope of 4 mm outside diameter (Olympus Visera Pro system, Center Valley, PA America, with OTV-S7Pro camera and CLV-S40Pro light source). This intraoperative analysis allowed for the identification of the interface between the likely neoplastic/dysplastic and likely healthy areas, i.e. NBI margin. Next, the maxillofacial surgeon performed multiple biopsies of both the MM and the NBI margins. For each patient, from 2 to 6 bioptic samples were obtained. The NBI margins were then classified as overlapping, external, or internal, compared to the MM. In case of overlap between the MM and NBI margins, only one biopsy was performed (Fig. 1).

### Histopathological analysis and pathologic surgical margins assessment

Hematoxylin and eosin (H&E) stained frozen sections of the MM margins were intraoperatively analyzed by a pathologist as part of the standard protocol to drive the surgical intervention. All surgical samples, including the tumor, the MM and NBI margins, were then analyzed after tissue processing by two pathologists (NF and MM), as shown in Fig. 1a. Specifically, all cases were classified and graded following the latest World Health Organization criteria [15]. Pathologic staging was assessed according to the current TNM staging system [16]. The presence of tumor infiltrating lymphocytes (TILs) was assessed as described [17]. The MM and NBI surgical margins were defined as positive in the presence of OSCC and/or dysplasia; otherwise, they were marked as negative.

### Immunohistochemistry and microvessel density analysis

MD was investigated by immunohistochemistry (IHC) in the MM and NBI surgical margins. Representative 4-μm-thick sections were cut from the MM, NBI, and tumor blocks and subjected to IHC using pre-diluted antibodies against CD34 as previously described [18]. Positive and negative controls were included in each slide run. Briefly, the protocols use an automated staining system (Dako Omnis) and anti-human prediluted antibodies [19]. Protein expression was analyzed in all different samples by two independent pathologists (NF and SF). Discordant results were resolved during dedicated consensus sessions. Sections were first observed at low magnification (40x) to identify the areas with the higher concentration of vessels. Then, the vessels count was performed at 200x by means of a customized digital image analysis algorithm using the Aperio CS2 instrument (Leica Microsystems Srl) [20]. The MD value was expressed as a percentage. Each CD34-positive structure (round, oval, and irregular) separated from other profiles or tissue elements was counted as a single vessel, regardless of the presence of a clear lumen.

### Statistical analysis

Data were analyzed using Prism 4.0 (GraphPad Inc., La Jolla, CA, USA). Differences among sample groups were analyzed using the unpaired Student’s t-test as previously described [21]. The association between positive margins was evaluated by Fisher’s exact test according to the classification proposed by Piazza and collaborators [22]. Statistical significance was assumed for a probability value (p) less than 0.05.

## Results

Sixteen patients (10 males and 6 females) who underwent surgery for OSCC were included in this study (age 23 to 92 years old, mean 68 years). Tumor sites included the tongue (*n* = 6), lower alveolar ridge/mandible (*n* = 3), hard palate (*n* = 2), cheek (*n* = 2), floor of mouth (*n* = 2), and upper alveolar ridge/maxilla (*n* = 1). Clinicopathologic data are summarized in Table 1.

**Table 1:** Demographic and clinicopathologic features of the study group

Features	Number of cases (%)
Sex	
Male	10 (62.5)
Female	6 (37.5)
Age	
Mean	68.25
Smoking	
Yes	3 (19)
No	4 (25)
Ex smoker	9 (56)
Alcohol	
Yes	12 (75)
No	3 (19)
Ex drinker	1 (6)
Site	
Tongue	6 (37.5)
Mandible	3 (19)
Palate	2 (12.5)
Cheek	2 (12.5)
Floor of the mouth	2 (12.5)
Maxilla	1 (6)
T Staging	
T1	6 (37.5)
T2	5 (31.25)
T3	1 (6.25)
T4	4 (25)
N Staging	
Nx	3 (18.75)
N0	7 (43.75)
N1	3 (18.75)
N2	3 (18.75)
Grading	
G1	2 (12.5)
G2	13 (76.4)
G3	1 (6.25)
Vascular invasion	
Yes	0
No	9 (56.25)
Perineural invasion	
Yes	5 (31.25)
No	11 (68.75)

### Integration of MM and NBI margins is superior to MM and NBI alone

**Fig. 2:**
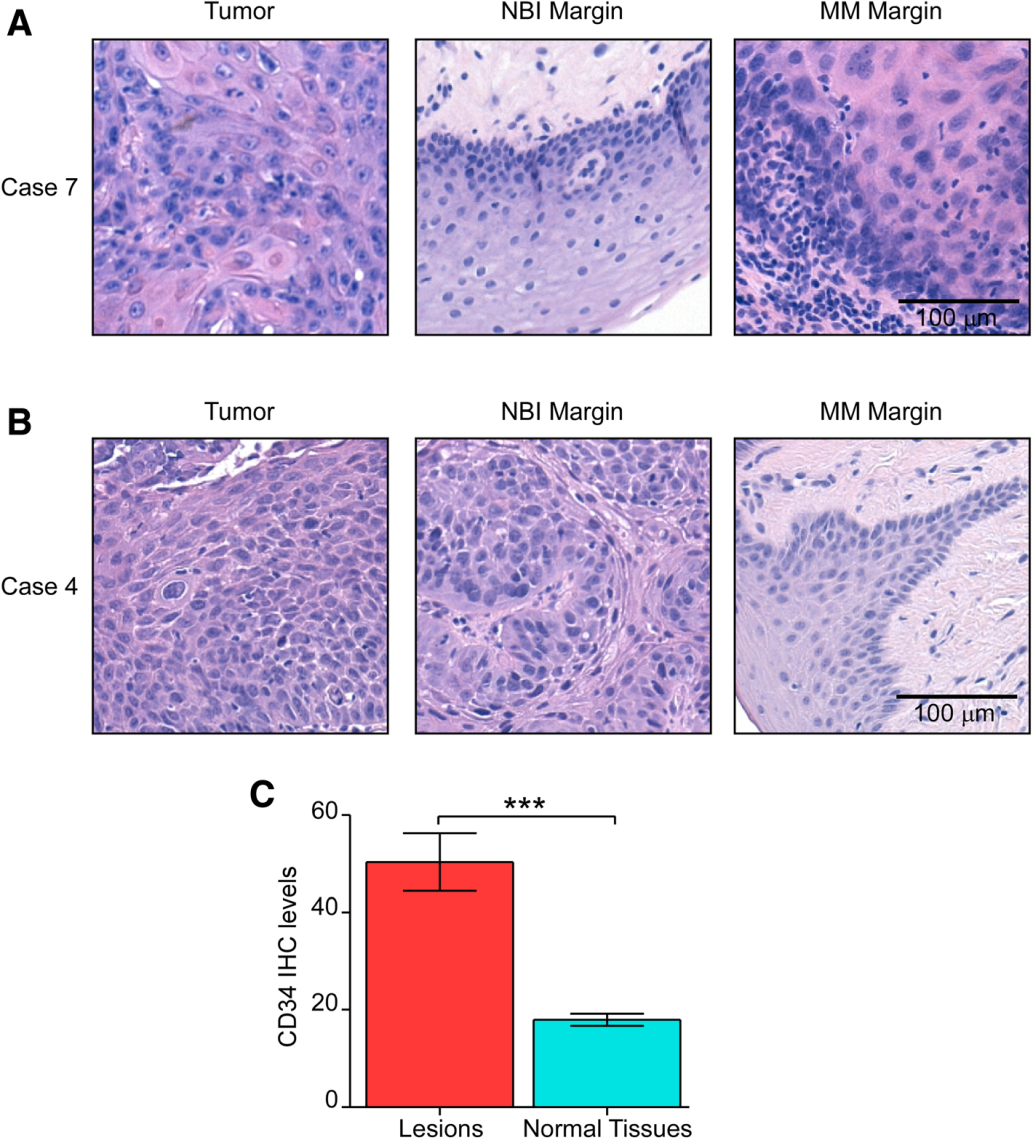
**a** Representative histological micrographs of the primary tumor, NBI and MM margins in a case (#7) where NBI margins are negative and a MM shows low-grade dysplasia. **b** Representative histological micrographs of the primary tumor, NBI and MM margins in a case (#4) were an NBI margin is positive for high-grade dysplasia, while MM are negative. Original magnification is 100X. **c** CD34 protein levels in positive (red) and negative (cyan) margins. *p* < 0.0001 by unpaired Student’s t-test

Taken together, 104 specimens were analyzed, including 16 (15.4%) tumors, 34 (32.7%) MM, 34 (32.7%) NBI margins, and 20 (19.2%) MM-NBI overlapping margins (Fig. 1b). The NBI margins were closer to the lesion in 17 (50%) cases (Fig. 1b) compared to the MM assessment. However, this method showed no propensity to allow for a more conservative resection, given that in the same number of margins (*n* = 17, 50%) was the MM the more conservative approach. Furthermore, this heterogeneity was irrespective of the tumor site and was not present at a single-patient level. At the histological examination, the margins collected with the MM intraoperative assessment revealed dysplasia in 3 (8.8%) cases and OSCC in 1 (2.9%) case, while 30 (88.2%) samples were negative as represented in Table 2 and Fig. 2a. The analysis of the NBI margins showed dysplasia and OSCC in 2 (5.9%) and 1 (2.9%) cases; respectively, while 31 (91.2%) margins were negative, as confirmed by histological examination (Table 2 and Fig. 2b). Among the 20 overlapping MM-NBI margins, 2 (10%) cases were positive. In particular, positive margins showed a significant association with thick and thin non-keratinized epithelial cells [23] (*p* = 0.027). These data suggest that the intraoperative integration of MM and NBI analysis might allow for a more conservative excision of OSCC compared to each of the two methods alone.

**Table 2:** Demographic, clinicopathologic characteristics, and surgical margins status of the patients included in this study. NBI, narrow-band imaging; MM, macroscopic margin, AU, alcohol units; n.a., not available; RT, radiotherapy; CT, chemotherapy. When NBI and MM were overlapping, only one biopsy was performed

Case	Age (range)	Smoking	Alcohol	Site	Piazza et al. Classification [23]	Staging TNM	Grading	Lympho-vascular/Perineural invasion	Sample 1 NBI/MM	Sample 2 NBI/ MM	Sample 3 NBI/ MM	Sample 4 NBI/ MM	Adjuvant Therapy
1	50–60	No	Yes (2 AU/die)	Lateral Tongue	2b	T1Nx	G2	No/No	–	−/−	–	–	No
2	70–80	Ex	Yes (2 AU/die)	Floor of mouth	2a	T2 N0	G2	No/Yes	−/−	–	n.a.	n.a.	RT
3	50–60	Yes	Yes (2 AU/die)	Ventral Tongue	2a	T2 N0	G2	No/Yes	−/−	−/−	n.a.	n.a.	RT
4	50–60	Ex	Yes (1 AU/die)	Floor of mouth	2a	T1(m)Nx	n.a.	No/No	+/−	−/−	−/−	n.a.	CT + RT
5	50–60	Ex	Yes (1 AU/die)	Maxilla/Alveolar Mucosa	1	T4aNx	G1	No/No	−/−	−/−	n.a.	n.a.	No
6	> 80	No	Yes (1 AU/die)	Mandible/Alveolar Mucosa	1	T4aN0	G1	No/No	−/−	−/−	−/−	−/−	No
7	60–70	Ex	Yes (1 AU/die)	Lateral Tongue	2b	T1 N1(E-)R1	G2	No/No	n.a.	−/−	–	−/+	CT + RT
8	> 80	No	No	Cheek	2b	T2N1R0	G2	No/Yes	n.a.	−/+	−/−	–	No
9	> 80	No	Ex Drinker	Hard Palate	1	T4aN2b (E-) R0	G2	No/No	–	–	–	−/−	No
10	70–80	Ex	No	Mandible/Retromolar Trigone	2b	T2 N1 (E-) R0	G3	No/No	+/−	–	−/−	−/−	RT
11	< 30	Ex	Yes (1 AU/die)	Lateral Tongue	2b	T2N2b	G2	No/Yes	+/+	−/−	+	−/−	CT + RT
12	60–70	Ex	Yes (1 AU/die)	Mandible/Alveolar Mucosa	1	T2 N0R0	G2	No/Yes	–	−/−	–	–	No
13	> 80	Yes	No	Lateral Tongue	2b	T1N2b	G2	No/No	–	–	–	−/+	No
14	> 80	Ex	Yes (1 AU/die)	Cheek	2b	T1 N0	G2	No/No	+	−/−	−/−	−/−	No
15	70–80	Ex	Yes (1 AU/die)	Hard Palate	1	T4aN0	G2	No/No	–	−/−	−/−	−/−	No
16	50–60	Yes	Yes (1 AU/die)	Floor of mouth	2a	T1 N0	G2	No/No	–	−/−	n.a.	−/−	No

### High levels of microvessel density are related to positive mucosal margins irrespective of the method used for their assessment

MD has been investigated in 83 margins and matched OSCCs. This analysis showed significantly high CD34 levels in pathological margins compared to the normal ones (*p* < 0.0001, Fig. 2c). This observation was unrelated to the intraoperative method of surgical margins assessment (i.e. MM and NBI). Internal and external margins didn’t show a statistically significant different MD, akin to the tumor site.

## Discussion

The use of new technologies to investigate tumor behavior and microenvironment is of great interest in this era of precision medicine. Several studies unraveled the role of molecular biomarkers for the diagnostic and therapeutic process in patients with OSCC [24–26]. The application of “biologic endoscopy” to intraoperative surgical procedures represents another step forward towards the realization of the potentials of customized surgery [9, 10, 27, 28]. Autofluorescence detection and NBI technology have already been tested in the definition of resection margins in OSCC and demonstrated to be reliable and cost-effective [9, 29]. Poh et al. [29] described the ability of autofluorescence to identify malignant and pre-malignant lesions. Tirelli et al. [10] reported an overall diagnostic gain of 8.5% using NBI, allowing a better definition of the tumor extension. They observed adequate resection margins in 74.2% of cases. Moreover, a resection enlargement of 11 ± 3 mm was performed consequently for intraoperative NBI evaluation [9], which revealed moderate dysplasia and cancer in 25 and 75% of samples respectively.

In this study, we performed a comparison between the mucosal margins assessment by MM and NBI, using their histological counterparts as "gold standard". Overall, we have observed that 50% of NBI margins were external or internal to the traditional surgical (i.e. MM) ones. These results confirm previous observations that NBI margins are usually wider than MM margins [9]. Interestingly, we observed that in approximately 30% of cases the NBI technology coupled with traditional surgical assessment is able to reduce the extent of the resection, as confirmed by the histological analysis. Moreover, NBI and MM specimens revealed 2 and 3 mild dysplasia, respectively. In particular, positive margins were significantly localized in thick and thin non-keratinized epithelia with a low papillary density [23]. These data confirm the safety of the NBI technique and provide previously unavailable data that the integration of MM and NBI margins is superior to MM and NBI alone in OSCC surgical management [23, 30].

There are several lines of evidence that the activation of neoangiogenesis pathways represents a founder molecular event in OSCC initiation and progression [31]. Previous studies have demonstrated that high levels of MD are associated with a more aggressive clinical course in head and neck cancers [31–35]. In the present study, MD was quantified by the measurement of the areas lined by elements expressing CD34, which is a transmembrane protein encoded by the homonymous gene located at chromosome 1q. Taken together, we detected significant higher levels of MD in the positive margins compared to the normal mucosa. In several solid tumors, neoangiogenesis carries heavy traffic of non-malignant cells, especially B and T lymphocytes. These data confirm crucial role of the immune surveillance in head and neck cancer [36, 37].

Here, we evaluated the surgical margins status in OSCC by means of NBI endoscopy and the pathological identification of neoangiogenesis and intratumor immune response. This pilot study highlights that the surgical and NBI margins are comparable in terms of reliability. This notion, however, should be considered in the context of the small sample size investigated in the present work. An intrinsic limitation of this study is represented by absence of deep margin assessment, given that the NBI technology allows only for the evaluation of the perimetral margins. Further prospective studies embracing larger cohorts of patients are warranted to define the operational implications of our observations. This would lead to standardized intraoperative employment of this novel integrated strategy.

## Conclusion

The integration of the traditional MM assessment with the NBI technology can allow for more conservative surgical interventions in OSCC.

## Abbreviations

ENE: Extranodal extension
ENT: Ears, nose and throat
H&E: Hematoxylin and eosin
IHC: Immunohistochemistry
MD: Microvessel density
MM: Macroscopic margins
NBI: Narrow band imaging
OSCC: Oral squamous cell carcinoma
TILs: Tumor infiltrating lymphocytes
